# Supplementary material for: Epigenetic Silencing of miR-33b Promotes Peritoneal Metastases of Ovarian Cancer by Modulating the TAK1/FASN/CPT1A/NF-κB Axis
Source: Cancers (Basel). 2021 Sep 24;13(19):4795. doi: 10.3390/cancers13194795 (PMC8508465; doi:10.3390/cancers13194795)
Supplement: Supplementary file 1 [file cancers-13-04795-s001.zip › supplementary material/cancers-1367272-supplementary.pdf]

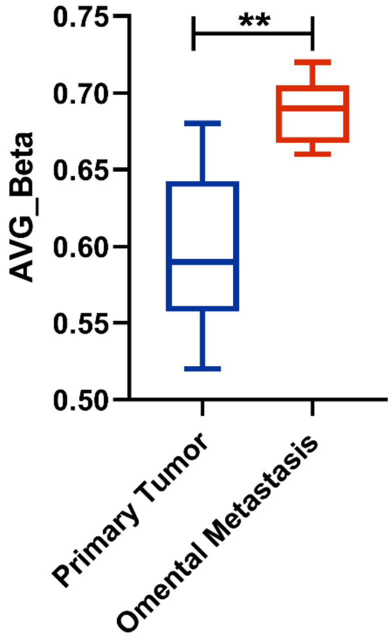

Figure S1. Boxplot comparing DMP methylation levels between primary and metastatic ovarian tumor tissues (N=6). The average DMP methylation levels were 0.597 and 0.688 in the primary and metastatic tumor groups, respectively. ** $p < 0.01$, student t test.

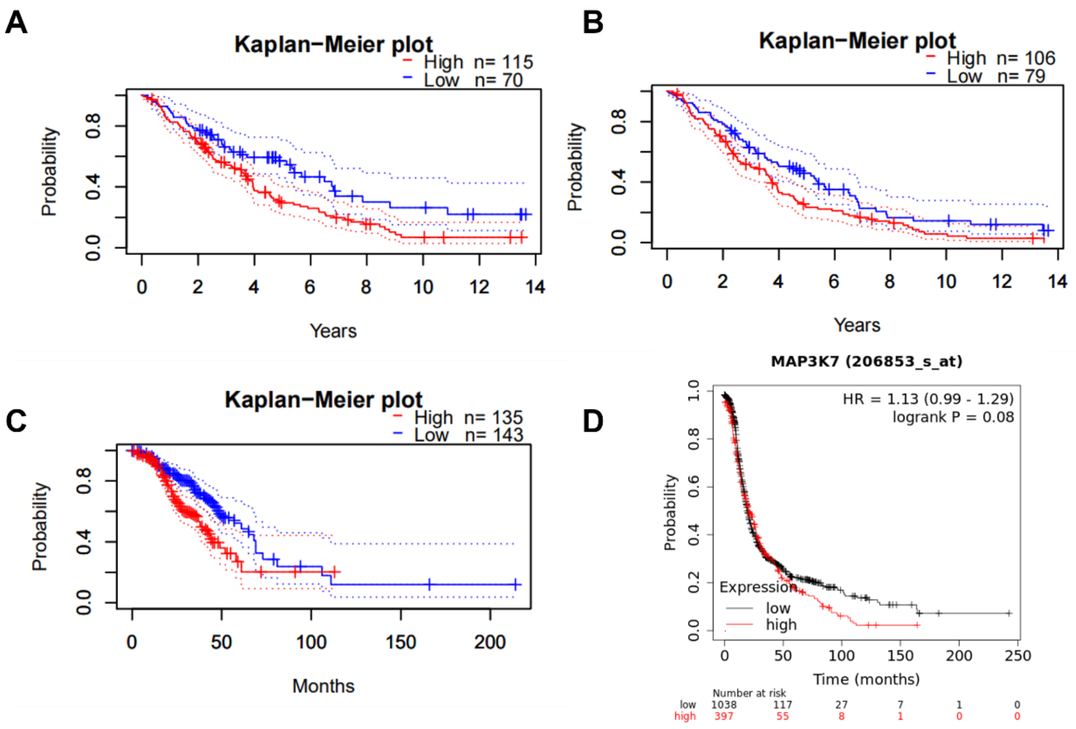

Figure S2. Prognostic value of TAK1 expression on survival rates in ovarian cancer using GEO and TCGA data. **(A,B)** The impact of TAK1 expression on **(A)** overall survival (HR=1.94, P=0.009) and **(B)** disease-free survival rate (HR=1.62, P=0.046) in a cohort of 185 ovarian cancer patients from GSE26712 dataset. **(C)** The impact of TAK1 expression on overall survival (HR=1.42, P=0.038) in a cohort of 278 ovarian cancer patients from GSE9891 dataset. **(D)** The impact of TAK1 expression on overall survival (HR=1.13, P=0.08) in a cohort of 1435 ovarian cancer patients from TCGA database.

Table S1. Primers for methylation analysis

Analysis	Sequence[a]
MSP	F: 5'- TGTGTCGAATTTGTTCGTC -3'<br>R: 5'- ACGCCCAAAAAACGTAAA -3'
USP	F: 5'- AATTGTGTTGAATTTGTTTGTT -3'<br>R: 5'- ACACCCAAAAAACATAAACAC -3'
Pyrosequencing	F: 5'- AGGTTGGATTTTTGATTTTTGATAGGTATA -3'<br>R: 5'- CCATTTTTCAAAAAAAACATACTTATCT -3'<br>(With Biotin)<br>SQ: 5'-AGGTATATTATTATAGAGGGG -3'

a: F=Forward primer; R=Reverse primer; SQ=Sequencing primer

Table S2. Kits and reagents used in this study.

Kits/Assays	Companies
Cleanascite™ Lipid Removal Reagent	Biotech Support Group, Monmouth Junction, NJ, USA
Extracellular O2 Consumption Assay Kit	Abcam, Cambridge, MA, USA
Fatty Acid Oxidation Assay Kit	Abcam, Cambridge, MA, USA
Luminescent ATP Detection Assay Kit	Abcam, Cambridge, MA, USA
The Lipolysis Assay Kit (Colorimetric)	Abcam, Cambridge, MA, USA
Triglyceride Assay Kit	Abcam, Cambridge, MA, USA
Etomoxir (Cat. No. 4539)	Tocris Bioscience, Minneapolis, MN, USA
Orlistat (Cat. No. 3540)	Tocris Bioscience, Minneapolis, MN, USA

Table S3. Antibodies used for Western blotting.

**Antibodies**	**Company**	**Dilution**
TAK1 (D94D7) mAb #5206	Cell Signaling Technology, USA	1:1000
Phospho-TAK1 (Ser412) Antibody #9339	Cell Signaling Technology, USA	1:1000
Phospho-IκBα (Ser32) (14D4) mAb #2859	Cell Signaling Technology, USA	1:1000
IκBα (L35A5) mAb (Amino-terminal Antigen) #4814	Cell Signaling Technology, USA	1:1000
Phospho-IKKα/β (Ser176/180) (16A6) mAb #2697	Cell Signaling Technology, USA	1:1000
IKKβ (D30C6) mAb #8943	Cell Signaling Technology, USA	1:1000
IKKα (3G12) mAb #11930	Cell Signaling Technology, USA	1:1000
Fatty Acid Synthase (C20G5) mAb	Cell Signaling Technology, USA	1:1000
CPT1A (D3B3) mAb # 12252S	Cell Signaling Technology, USA	1:1000
GAPDH (D16H11) XP® mAb #5174	Cell Signaling Technology, USA	1:1000
IRDye® 680 RD/800 CW Goat anti-Mouse IgG Secondary Antibody	LI-COR Biosciences, USA	1:15000
IRDye® 680 RD/800 CW Goat anti-Rabbit IgG Secondary Antibody	LI-COR Biosciences, USA	1:15000
