# Supplementary figures and images for: Epigenetic Silencing of miR-33b Promotes Peritoneal Metastases of Ovarian Cancer by Modulating the TAK1/FASN/CPT1A/NF-κB Axis
Source: Cancers (Basel). 2021 Sep 24;13(19):4795. doi: 10.3390/cancers13194795 (PMC8508465; doi:10.3390/cancers13194795)

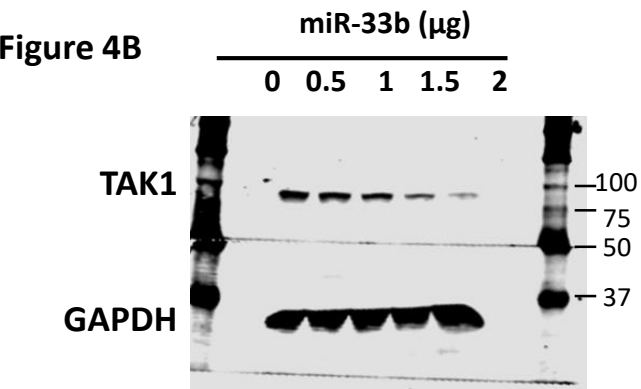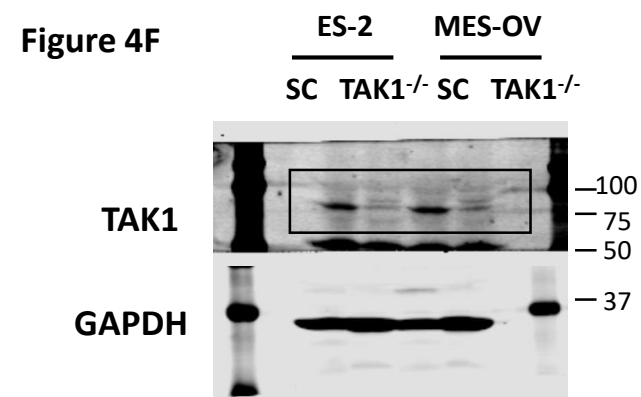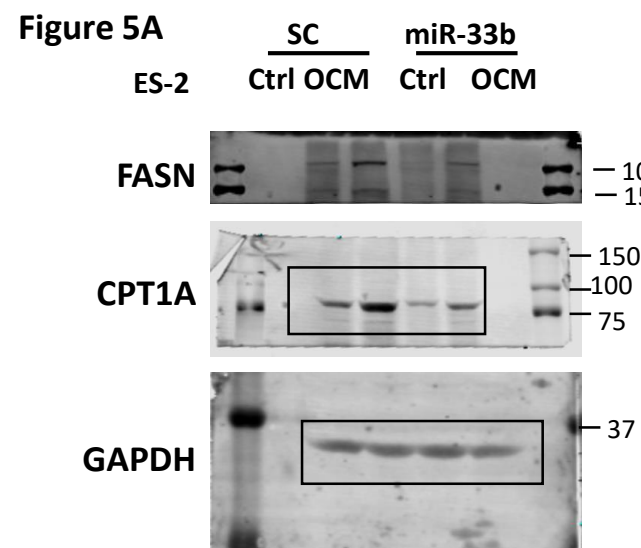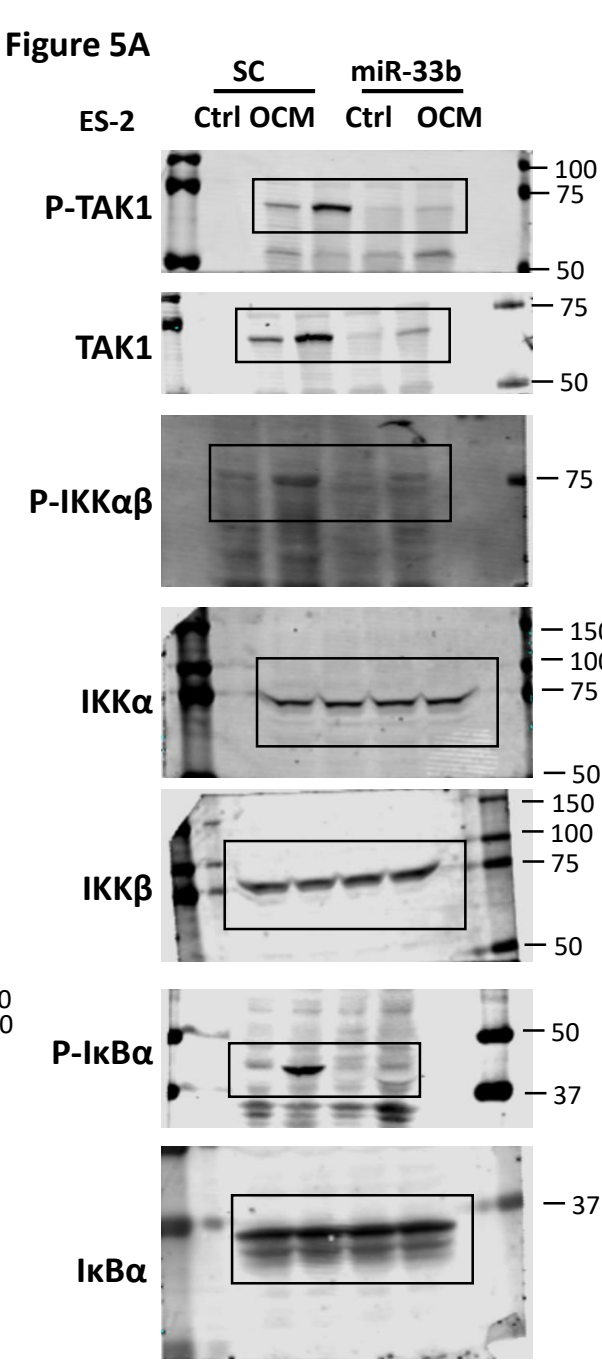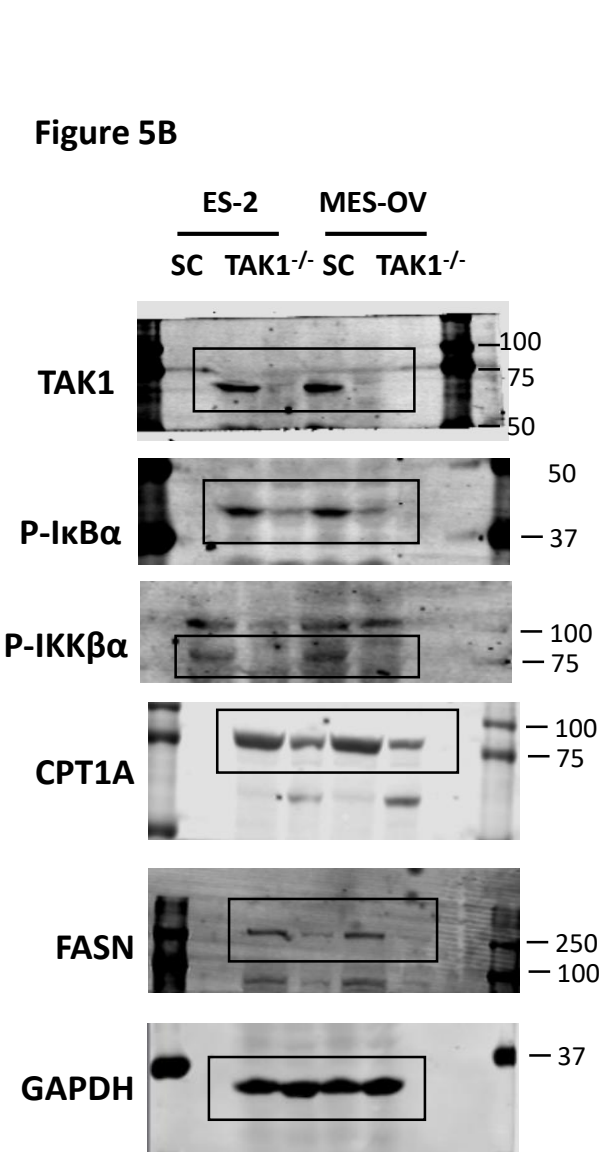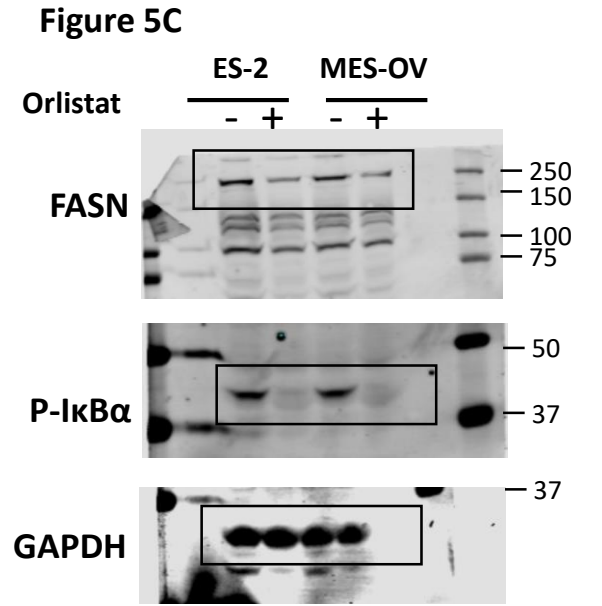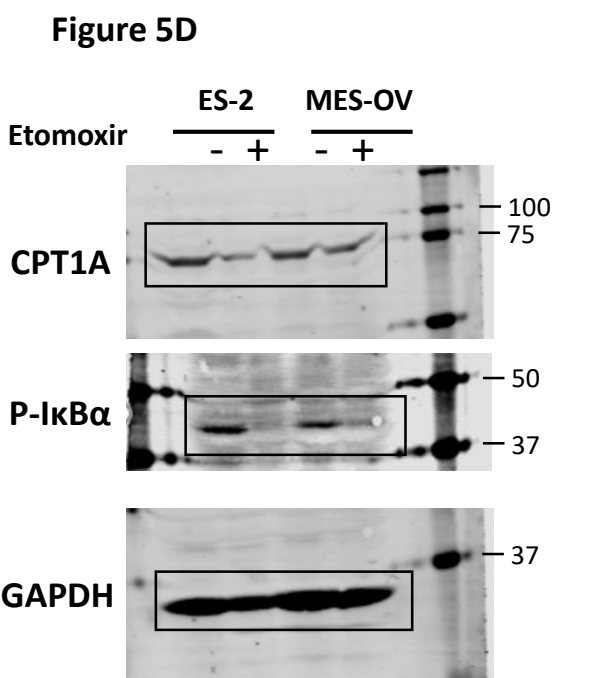

Supplement: Supplementary file 1 [file cancers-13-04795-s001.zip › supplementary material/File S1.pdf]
